# Ability of a single meal composition in changing postprandial inflammatory responses

**DOI:** 10.1186/1758-5996-7-S1-A230

**Published:** 2015-11-11

**Authors:** Milena Monfort Pires, Amanda Rabello Crisma, Sandra Roberta Gouvea Ferreira

**Affiliations:** 1 Faculdade de Saúde Pública, Universidade de São Paulo, São Paulo, Brazil

## Background

Modern Western diet contributes to weight gain, an important cardiometabolic risk factor. In contrast, the Mediterranean diet has been associated with cardioprotection. Whether small changes in dietary habits, including Mediterranean foods, could induce metabolic benefits is unknown.

## Objective

The effects of a Brazilian typical breakfast (BRAZ) and a modified breakfast with Mediterranean food (MOD) on postprandial metabolic profile and inflammatory genes were compared.

## Materials and methods

This crossover clinical trial included 80 overweight individuals who received one of two isocaloric breakfasts (BRAZ: coffee, whole fat milk, french bread, butter and mozzarella cheese; MOD: coffee, 1% fat milk, wheat bread, ricotta cream, olive oil and peanuts) for 4 weeks in a random order. After a 2-week washout, individuals received the other intervention. Before and after each intervention period, individuals underwent a fat tolerance test with breakfasts. Inflammatory markers were assessed by ELISA and gene expression by PCR array. Variables were compared by repeated measures ANOVA and correlations between inflammatory and dietary data using Pearson coefficient.

## Results

At the end of both interventions, participants (51.7±9.5 yrs.; body mass index of 30.5±4.2 kg/m2), did not change anthropometry, plasma glucose or triglycerides. Inflammatory markers showed that BRAZ and MOD interventions provoked, respectively, contrasting Results in fasting E-selectin (13.1±5.0 to 18.1±7.0 vs. 14.2±5.9 to 13.2±6.6 ng/mL), TNF-α (3.2±1.3 to 6.1±1.9 vs. 3.4±2.1 to 2.7±1.7 pg/mL), IFN-γ (1.5±0.6 to 2.7±0.7 vs. 2.0±0.9 to 1.8±1.0 pg/mL), IL-6 (2.3±0.8 to 5.7±1.9 vs. 3.1±2.0 to 2.2±1.5 pg/mL) and IL-8 (3.7±2.4 to 4.2±2.6 vs. 5.8±4.1 to 3.8±2.5 pg/mL), and also in postprandial responses to fat test (p diet <0.01). Changes in MUFA intake and changes in inflammatory markers were inversely correlated, while changes in saturated fat intake were directly correlated to IFN-γ and IL-6. After the BRAZ and MOD interventions, differences in postprandial relative gene expression of IL-1α (2.31 vs. 0.37), colony stimulating factor 2 (1.96 vs. 0.36) and E-selectin (2.43 vs 0.68), were observed.

## Conclusions

Modification of a single meal can improve cardiometabolic risk in the short-term by reducing low-grade inflammation. Changes in types of dietary fat may contribute to reduce postprandial inflammation. Our findings should motivate changes in eating habits in non-Mediterranean countries.

